# Whole-Genome Shotgun Sequence of Streptococcus agalactiae Sequence Type 176 Strain 3966RFQB from a Dairy Herd in Selangor, Malaysia

**DOI:** 10.1128/MRA.01618-18

**Published:** 2019-02-07

**Authors:** A. Bashir, Z. Zunita, F. F. A. Jesse, S. Z. Ramanoon, M. L. Mohd-Azmi

**Affiliations:** a Faculty of Veterinary Medicine, Universiti Putra Malaysia, Serdang, Selangor, Malaysia; b Department of Biological Sciences, Sule Lamido University, Kafin-Hausa, Jigawa State, Nigeria; University of Maryland School of Medicine

## Abstract

Streptococcus agalactiae, commonly known as group B streptococcus (GBS), is among the most implicated pathogens in bovine mastitis worldwide. Proper control measures can curb both economic and public health effects it may cause. Here, we report the sequenced genome of S. agalactiae sequence type 167 (ST167) strain 3966RFQB obtained from a bovine mastitis case at a dairy herd in Banting, Selangor, Malaysia (longitude 2.8121°N, latitude 101.5026°E).

## Abstract (precis)

Streptococcus agalactiae, commonly known as group B streptococcus (GBS), is among the most implicated pathogens in bovine mastitis worldwide. Proper control measures can curb both economic and public health effects it may cause.

## ANNOUNCEMENT

Streptococcus agalactiae, also commonly known as group B streptococcus (GBS), is one of the major causes of mastitis worldwide leading to high economic loss in the dairy industry (1). It is also an important human pathogen that can cause serious infections in infants and immunocompromised people, including the elderly and pregnant women (2). Many studies have shown a close relationship between streptococcal infection in many domestic animals and human infections caused by streptococcus (3). Proper control and preventive measures may certainly help in drastically reducing the rate of mastitis caused by S. agalactiae, thus improving quantity and quality of milk production and, most significantly, ensuring animal welfare and reducing public health threats (2). There are many treatment and control measures against mastitis, but these are clearly not able to contain the disease on farms (4). Vaccination can play an important role in mastitis control programs (5). The selection of potential vaccine candidates requires the identification of virulent proteins which can be able to stimulate immune response in the host organism. The basic rationale behind the identification of potential vaccine targets is the prediction of antigenic and virulence determinants between disease-causing organisms (6).

We sequenced the S. agalactiae sequence type 167 (ST167) strain 3966RFQB genome for the purpose of identifying immunogenic targets for vaccine developments against streptococcal mastitis.

S. agalactiae ST167 3966RFQB was recovered from a California mastitis test (CMT)-positive milk sample. For isolation, the samples were cultured on blood agar (Oxoid, Hampshire, England). Cultured plates were incubated aerobically for 24 hours at 37°C. The plates were then examined for growth and morphological features, such as colony size, shape, color, and hemolytic characteristics. Following isolation, S. agalactiae was characterized using Gram reaction, catalase test, and coagulase test. Finally, the analytical profile index (API) system (bioMérieux, France) was used for identification following the manufacturer’s guidelines.

Genomic DNA extraction from the cultured cells was carried out using the Wizard genomic DNA extraction kit (Promega, Wisconsin, USA) following the manufacturer’s guidelines at the Bacteriology Laboratory, Faculty of Veterinary Medicine, Universiti Putra Malaysia. The DNA samples were examined for good quality using the NanoDrop 2000c spectrophotometer (Thermo Fisher Scientific, Waltham, MA, USA). The DNA integrity was further checked using 1% agarose gel electrophoresis.

For the whole-genome sequencing, a paired-end library was constructed using a Nextera XT DNA library preparation kit (Illumina, San Diego, CA, USA) following the manufacturer’s guidelines. The library was sequenced on the MiSeq 2000 (Illumina) platform using 2 × 150 chemistry. BBDuk tool version 36 (http://jgi.doe.gov/data-and-tools/bbtools/) was used for adapter trimming, quality trimming, contaminant filtering, and read length filtering.

The sequence reads were *de novo* assembled by SPAdes version 3.9.0 (7). Ultimately, the genome was assembled into 33 large contigs, with an *N*_50_ value of 139,167 bp and the longest contig length of 471,672 bp. The genome is approximately 2.2 Mb in length, with a G+C content of 35.46% and an average coverage depth of 115.2×.

For genome annotation, 2,156 protein-coding genes were predicted using Prodigal version 2.60 (8). The functions of the protein-coding genes were predicted from their predicted sequences using BLASTN version 2.2.31+ (9) and HMMER version 3.0 (10) to enable searches from various databases. Finally, 44 tRNA genes were predicted using ARAGORN version 1.2.34 (11), and 3 rRNA genes were predicted using RNAmmer version 1.2 (12).

### Data availability.

The whole-genome shotgun project has been deposited at DDBJ/ENA/GenBank under the GenBank accession number QLZC00000000. The version described in this paper is the first version, QLZC01000000. The SRA data can be accessed using the accession number PRJNA476670.
