# Correction for Bashir et al., “Whole-Genome Shotgun Sequence of Streptococcus agalactiae Sequence Type 176 Strain 3966RFQB from a Dairy Herd in Selangor, Malaysia”

**DOI:** 10.1128/MRA.00331-19

**Published:** 2019-04-18

**Authors:** A. Bashir, Z. Zunita, F. F. A. Jesse, S. Z. Ramanoon, M. L. Mohd-Azmi

**Affiliations:** a Faculty of Veterinary Medicine, Universiti Putra Malaysia, Serdang, Selangor, Malaysia; b Department of Biological Sciences, Sule Lamido University, Kafin-Hausa, Jigawa State, Nigeria

## AUTHOR CORRECTION

Volume 8, no. 6, e01618-18, 2019, https://doi.org/10.1128/MRA.01618-18. Page 1: The author byline should appear as given in this correction.

